# Cold plasma pretreatment reinforces the lignocellulose-derived aldehyde inhibitors tolerance and bioethanol fermentability for *Zymomonas mobilis*

**DOI:** 10.1186/s13068-023-02354-8

**Published:** 2023-06-15

**Authors:** Xia Yi, Dong Yang, Xiaoyan Xu, Youjun Wang, Yan Guo, Meng Zhang, Yilong Wang, Yucai He, Jie Zhu

**Affiliations:** 1 National-Local Joint Engineering Research Center for Biomass Refining and High-Quality Utilization, Changzhou University, Changzhou, 213164 China; 2 Institute of Urban and Rural Mining, Changzhou University, Changzhou, 213164 China; 3 Changzhou Key Laboratory of Biomass Green, Safe & High Value Utilization Technology, Changzhou University, Changzhou, 213164 Jiangsu China; 4 School of Pharmacy, Changzhou University, Changzhou, 213164 Jiangsu China

**Keywords:** Bioethanol, *Zymomonas mobilis*, Cold atmosphere plasma (CAP), Genome resequencing, RNA-Seq sequencing

## Abstract

**Background:**

Lignocellulose-derived aldehyde inhibitors seriously blocked the biorefinery of biofuels and biochemicals. To date, the economic production of lignocellulose-based products heavily relied on high productivities of fermenting strains. However, it was expensive and time-consuming for the achievable rational modification to strengthen stress tolerance robustness of aldehyde inhibitors. Here, it aimed to improve aldehyde inhibitors tolerance and cellulosic bioethanol fermentability for the chassis *Zymomonas mobilis* ZM4 pretreated using energy-efficient and eco-friendly cold plasma.

**Results:**

It was found that bioethanol fermentability was weaker in CSH (corn stover hydrolysates) than that in synthetic medium for *Z. mobilis*, and thus was attributed to the inhibition of the lignocellulose-derived aldehyde inhibitors in CSH. Convincingly, it further confirmed that the mixed aldehydes severely decreased bioethanol accumulation through additional aldehydes supplementary assays in synthetic medium. After assayed under different processing time (10–30 s), discharge power (80–160 W), and working pressure (120–180 Pa) using cold atmosphere plasma (CAP), it achieved the increased bioethanol fermentability for *Z. mobilis* after pretreated at the optimized parameters (20 s, 140 W and 165 Pa). It showed that cold plasma brought about three mutation sites including *ZMO0694* (E220V), *ZMO0843* (L471L) and *ZMO0843* (P505H) via Genome resequencing-based SNPs (single nucleotide polymorphisms). A serial of differentially expressed genes (DEGs) were further identified as the potential contributors for stress tolerance via RNA-Seq sequencing, including *ZMO0253* and *ZMO_RS09265* (type I secretion outer membrane protein), *ZMO1941* (Type IV secretory pathway protease TraF-like protein), *ZMOr003* and *ZMOr006* (16S ribosomal RNA), *ZMO0375* and *ZMO0374* (levansucrase) and *ZMO1705* (thioredoxins). It enriched cellular process, followed by metabolic process and single-organism process for biological process. For KEGG analysis, the mutant was also referred to starch and sucrose metabolism, galactose metabolism and two-component system. Finally, but interestingly, it simultaneously achieved the enhanced stress tolerance capacity of aldehyde inhibitors and bioethanol fermentability in CSH for the mutant *Z. mobilis*.

**Conclusions:**

Of several candidate genetic changes, the mutant *Z. mobilis* treated with cold plasma was conferred upon the facilitated aldehyde inhibitors tolerance and bioethanol production. This work would provide a strain biocatalyst for the efficient production of lignocellulosic biofuels and biochemicals.

## Background

As a potential resource for bioethanol production, valorization of lignocellulosic biomass available in massive quantities significantly offered positive environmental impacts through lessening greenhouse gas and other pollutant emissions [1, 2]. It is a crucial step for pretreatment process in biorefinery to disrupt biomass recalcitrance [3–5]. However, the resulting furanic aldehydes [2-furaldehyde (furfural) and 5-hydroxymethyl-2-furaldehyde (HMF)], weak organic acids (acetic acid, formic acid and levulinic acid) and phenolic aldehydes (4-hydroxybenzaldehyde, syringaldehyde and vanillin) seriously inhibited cell growth and product accumulation for fermenting strains [6–14]. Therefore, to make lignocellulose utilization of bioethanol more competitive with fossil fuel, genetic modification of fermenting strains was carried out to efficiently tolerate the stress of particularly most toxic aldehyde inhibitors [15–17].

Of high ethanol productivity and flexible genetic manipulation feasibility, the ethanologenic strain *Zymomonas mobilis* ZM4 performed its great potential in the lignocellulosic biorefinery fields [18, 19]. However, well tolerating furanic acids and phenolic acids, *Z. mobilis* was sensitive to furanic and phenolic aldehydes [10, 20]. Many efforts had been performed to enhance inhibitor tolerance for *Z. mobilis*, such as directed gene evolution [21–24], gene recombination [25–30] and gene mutation [31]. However, the growing efforts were still tentative for the aldehyde inhibitors-tolerant strain *Z. mobilis*.

Plasma, a quasi-neutral ionized or partially ionized gas in electric discharge, comprised the varied charged particles, metastable particles, molecules, neutral atoms, particles and photons [32, 33]. According to particles temperature, plasma was classified into equilibrium and non-equilibrium [34]. As the non-equilibrium plasma, low temperature plasma (LTP), such as atmospheric and room temperature plasma (ARTP) and low vacuum and room temperature plasma (LVRTP), could minimize the damaging effects on organisms and thermolabile matrices when delivered at room temperature [35]. Although ARTP could be widely carried out to treat microorganism suspension under non-vacuum condition, mutagenesis operation of the industrial strains was largely limited for its low energy generation and longstanding processing time [36–38]. LVRTP, also known as cold atmospheric plasma (CAP) and abbreviated as cold plasma, artificially generated at room temperature under atmospheric pressure, had also showed a brilliant prospect on the genetic modification of fermenting strains for its potent energy-efficient and eco-friendly advanced oxidation capacity in industrial fields [39–44]. To be sure, it was also applied to convey stress tolerance [45, 46]. However, little was on aldehyde inhibitors tolerance and bioethanol production for *Z. mobilis* ZM4 using cold plasma.

The current study tried to enhance aldehyde inhibitors tolerance and bioethanol production in corn stover hydrolysates (CSH) using cold plasma. Here, it assayed processing time, discharge power and working pressure to establish the optimum parameters. Furthermore, genetic variation and transcriptional profiling were uncovered through genome resequencing-based single nucleotide polymorphisms (SNPs) and transcriptional sequencing. This study would provide a strain biocatalyst as the potential producer of cellulosic biofuels and biochemicals.

## Materials and methods

### Feedstock and reagents

The commercial enzyme cellulase purchased from Sigma-Aldrich (St Louis, MO, USA) was determined of the 235 FPU/mL of filter paper activity following the NREL protocols LAP-006 method [47]. All the other analytical grade chemicals were from China National Pharmaceutical Group Co., Ltd (Sinopharm).

### Strain culture

*Z. mobilis* ZM4 was cultured in corn stover hydrolysate (CSH) liquids or RM (Rich Medium) medium containing 20.0 g/L glucose, 2.0 g/L KH_2_PO_4_, and 10.0 g/L yeast extract. A 1.0 mL of seed cultures was inoculated in 100 mL fresh RM medium in a 250-mL flask without shaking at 30 °C for the single-factor assays after cold plasma pretreatment. Sampling was at a 4 h interval till 24 h. The other fermentation assays were carried out with a 10% inoculation. It was added 4-hydroxybenzaldehyde, syringaldehyde, vanillin, furfural and HMF with the corresponding concentration of CSH to assay the tolerance of aldehyde inhibitors. For genome resequencing and RNA-Seq sequencing assays, the fresh culture of *Z. mobilis* ZM4 was harvested from 100 mL cultures at 4 h for RNA isolation. All assays were carried out in triplicate.

### CSH pretreatment

Harvested from Lianyungang, Jiangsu province, China, in the fall of 2021, corn stover milled to a size < 3 mm was pretreated with dilute acid [48, 49]. Hydrolysis of the pretreated corn stover was carried out in 134 °C for 1 h. The pretreated corn stover contained 49.93% cellulose, 22.80% hemicellulose and 20.35% lignin determined by the protocol described in NREL/TP-510-42618 [50]. Corn stover hydrolysates contained 22.94 g/L glucose, 0.01 g/L 4-hydroxybenzaldehyde, 0.55 g/L syringaldehyde, 0.08 g/L vanillin, 0.16 g/L furfural and 0.75 g/L HMF.

### Cold plasma pretreatment

Processed with cold plasma generated from the radio frequency power supply (13.56 MHz) using helium as working gas [51], *Z. mobilis* ZM4 streaked on the fresh RM plates cultured at 30 °C overnight was placed in the chamber of a cold plasma modification apparatus. The test parameters were covered as follows: processing time 10–30 s, discharge power 80–160 W and working pressure 120–180 Pa. After processed, the treated samples and the un-treated controls were re-activated by streaking on the fresh medium before fermentation assays.

### Genome resequencing

To confirm the genetic changes for *Z. mobilis* ZM4 pretreated with cold plasma under the optimum parameters, genome resequencing-based single nucleotide polymorphisms (SNPs) were performed by Beijing Novogene Bioinformatics Technology Co., Ltd (China).

Genomic DNA was quantified using Qubit^®^ 2.0 Fluorometer (Thermo Scientific) after isolated according to the SDS method [52]. It generated sequencing libraries using the NEBNextR Ultra^™^ DNA Library Prep kit for Illumina (NEB, MA, USA) following the manufacturer’s recommendations. After purified with AMPure XP system and analyzed on Agilent 2100 Bioanalyzer (Aligent, Santa Clara, CA), it sequenced the whole genome of *Z. mobilis* ZM4 using Illumina NovaSeq PE150. The original data derived from high-throughput sequencing were transformed into raw sequenced reads using base calling of CASAVA software before stored in FASTQ format. BWA software (version 0.7.8) and SAMTOOLS software (version 0.1.18) were separately used to map the Reads to the reference sequence of the wild *Z. mobilis* ZM4 and count the coverage of the reference sequence to the Reads [53, 54]. SNPs (single nucleotide polymorphisms)/InDel (insertion and deletion) analysis and SV (structural variation) analysis were carried out using SAMTOOLS software (version 0.1.18) and BreakDancer software (version 1.4.4), respectively [55]. To show reads coverage, SNPs distribution and InDel information, the online Circos software (version 0.64) was used to present the variation map of the whole genome [56]. The raw data of SNPs were submitted in European Variation Archive (EVA) [57]. For genome sequencing, besides the prepared genomic DNA samples pre-checked repeatedly, it also used DeconSeq (http://deconseq.sourceforge.net/) to eliminate the false signals from the potential bacterial or fungi contaminations before the assembly and the downstream analysis.

### RNA sequencing

Following the manufacturer’s instructions, the total RNA was isolated from *Z. mobilis* ZM4 samples using TRIzol^®^ reagent (Invitrogen, Carlsbad, CA, USA). The RNA was purified with the NucleoSpin RNA clean-up kit (Macherey–Nagel, Düren, Germany) and qualified with RIN (RNA Integrity Number) ≥ 7.0 and the ratio of 23S rRNA: 16S rRNA ≥ 15: 1 using Bioanalyzer 2100 (Aligent, Santa Clara, CA) before kept at −80 °C.

RNA-Seq sequencing assays were carried out by CapitalBio Technology Co., Ltd, Beijing, China. It quantified the initial concentration of the total RNA as 0.1–1.0 μg using Qubit RNA assay kit following the manufacturer’s instructions. The fragmented and primed mRNA from the rRNA depleted total RNA was used as the templates to synthesize the first strand of cDNA. Using dA-Tailed and ligating adaptor cDNA as the templates, PCR products were quantified with the Qubit DNA HS assay kit and qualified with 2100 Bioanalyzer chip. RNA-Seq sequencing assays were carried out on the Illumina NovaSeq 6000 platform. It used FastQ Screen (https://www.bioinformatics.babraham.ac.uk/projects/fastq_screen/) to screen contamination. HTSeq (high-throughput sequencing), a Python framework, was used to calculate gene counts of mRNA [58]. The differentially expressed mRNA was determined with DESeq [59]. Gene Ontology (GO) and KEGG pathway analysis were separately used DAVID (Database for Annotation, Visualization, and Integrated Discovery) and IPA (Ingenuity pathway analysis) software with *p* < 0.05. Here, differentially expressed genes (DEGs) was defined as an absolute value of log_2_ ratio ≥ 1.0, and the significant DEGs were required an additional *p* ≤ 0.05.

### HPLC analysis

Glucose and ethanol were determined at 55 °C and 0.6 mL/min using 5.0 mM H_2_SO_4_ as mobile phase using Thermo Scientific^™^ Dionex^™^ Ultimate^™^ 3000 high performance liquid chromatography (HPLC) equipped with a refractive index detector ERC RefractoMax 520 (Thermo Scientific, Waltham, MA, USA) and a Aminex HPX-87H column (Bio-Rad, Hercules, CA, USA). Furanic aldehydes and phenolic aldehydes were determined according to the previous methods [10, 60].

## Results

### Bioethanol fermentability in CSH weaker than that in synthetic medium for *Z. mobilis*

**Fig. 1:**
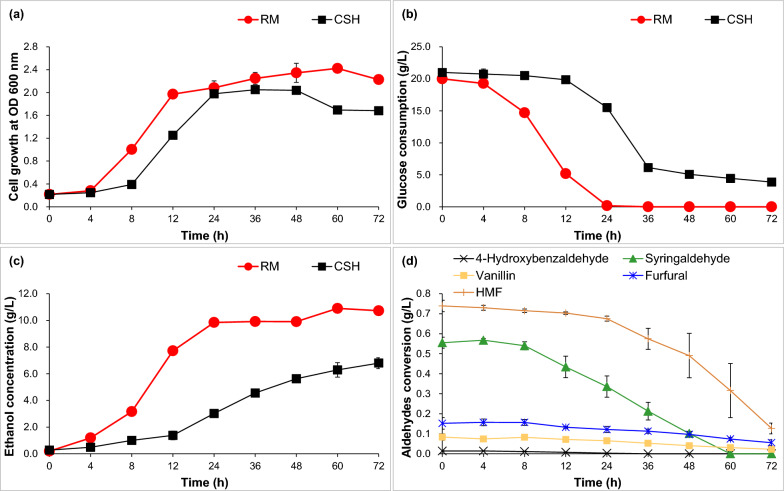
Ethanol fermentability in RM synthetic medium and CSH for *Z. mobilis* ZM4. **a** Cell growth; **b** glucose consumption; **c** ethanol concentration; **d** aldehyde conversion in CSH

Here, it compared bioethanol fermentability in CSH and RM synthetic medium for *Z. mobilis* ZM4 (Fig. 1). Cell growth in RM was higher by 12.57%, 61.18% and 36.52% separately at 4, 8 and 12 h than that in CSH (Fig. 1a). The glucose consumption in RM medium was 65.48%, 90.66% and 92.29% more than that in CSH (Fig. 1b). Bioethanol concentration in RM medium was 59.46%, 68.42% and 82.17% more than that in CSH (Fig. 1c). Interestingly, 0.01 g/L 4-hydroxybenzaldehyde, 0.55 g/L syringaldehyde and 0.08 g/L vanillin in CSH were degraded by 79.36% at 24 h, 81.76% at 48 h and 73.05% at 72 h, respectively (Fig. 1d). However, 0.16 g/L furfural and 0.75 g/L HMF were degraded by 63.66% and 82.90% at 72 h, respectively. Therefore, bioethanol fermentability in CSH was obviously weaker than that in RM synthetic medium for *Z. mobilis*, and aldehyde inhibitors were predicted as one of the main bottlenecks for the efficient production of bioethanol using CSH.

### Aldehyde inhibitors in CSH weakened bioethanol fermentability

**Fig. 2:**
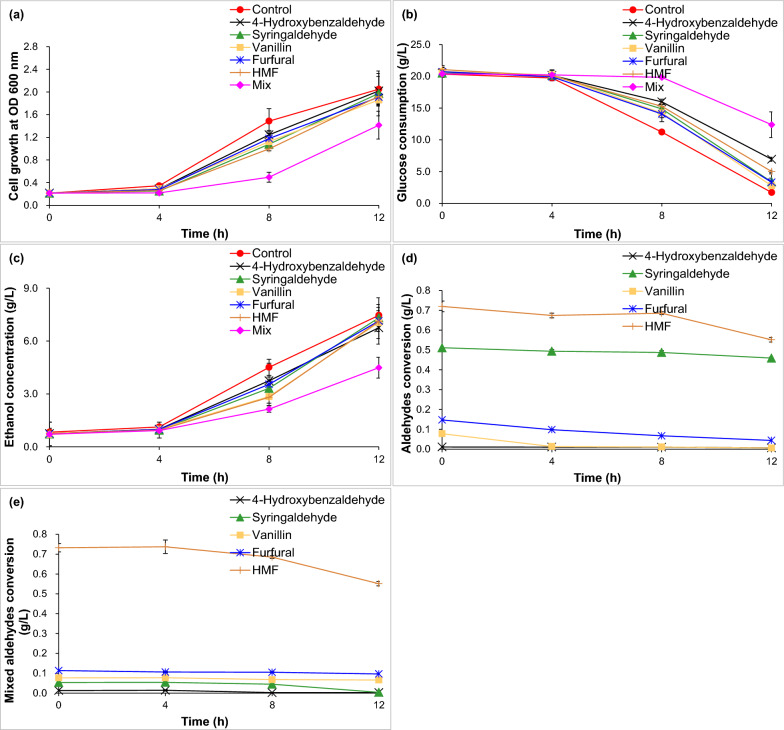
The effect of aldehyde inhibitors on ethanol fermentability for *Z. mobilis* ZM4. **a** Cell growth; **b** glucose consumption; **c** ethanol concentration; **d** aldehyde inhibitors conversion; **e** mixed aldehyde conversion

In order to verify aldehyde inhibitors in CSH as one of the blocks for bioethanol fermentability, it carried out fermentation assays in synthetic RM medium by adding aldehyde inhibitors of the corresponding concentration in CSH (Fig. 2). Compared with the control, it found that cell growth was inhibited by 15.90%, 26.98%, 15.13%, 19.56%, 24.66% and 36.71% at 4 h for 0.01 g/L 4-hydroxybenzaldehyde, 0.55 g/L syringaldehyde, 0.08 g/L vanillin, 0.16 g/L furfural, 0.75 g/L HMF and the above mixed aldehydes and by 16.23%, 27.40%, 24.35%, 20.63%, 33.00% and 66.61% at 8 h (Fig. 2a). Glucose consumption was inhibited by 44.51%, 43.26%, 12.94%, ND (not detected), ND, and 75.05% at 4 h and by 50.59%, 38.18%, 28.34%, 27.17%, 36.70% and 93.96% at 8 h (Fig. 2b). Bioethanol concentration was inhibited by 11.32%, 15.85%, 10.93%, 11.79%, 15.62% and 18.24% at 4 h; by 16.89%, 26.43%, 36.84%, 21.39%, 37.78% and 52.54% at 8 h (Fig. 2c). It indicated that the inhibitory intensity of the mixed aldehyde inhibitors was the highest for cell growth, glucose consumption and ethanol accumulation.

Figure 2d illustrates the conversion of aldehydes. It indicated that 0.08 g/L vanillin was degraded by 82.60%, 85.73% and 92.67% at 4, 8, and 12 h, respectively. 0.16 g/L furfural was separately degraded by 33.36%, 54.44% and 69.89%. However, no degradation of 0.75 g/L HMF, 0.01 g/L 4-hydroxybenzaldehyde and 0.55 g/L syringaldehyde were found at 4 and 8 h, and it separately determined 23.37%, 40.79% and 10.19% of degradation at 12 h. Equally, when five aldehydes were mixed together, 0.01 g/L 4-hydroxybenzaldehyde was preferentially degraded, followed by 0.55 g/L syringaldehyde (Fig. 2e). Totally, 0.01 g/L 4-hydroxybenzaldehyde, 0.55 g/L syringaldehyde, 0.08 g/L vanillin, 0.16 g/L furfural and 0.75 g/L HMF were degraded at 12 h by 81.89%, 93.28%, 14.71%, 14.76% and 24.74%, respectively.

Overall, it certainly confirmed that the mixture of aldehyde inhibitors with the corresponding concentration of the CSH obviously blocked bioethanol fermentability.

### Cold plasma pretreatment for *Z. mobilis*

In order to acquire a robust strain with the ability to tolerate aldehyde inhibitors of lignocellulosic hydrolysates, *Z. mobilis* ZM4 was treated using cold plasma as the mutagenesis tool to increase bioethanol production. Here, it carried out pretreatment assays of cold plasma under the different processing time, discharge powder and working pressure.

**Fig. 3:**
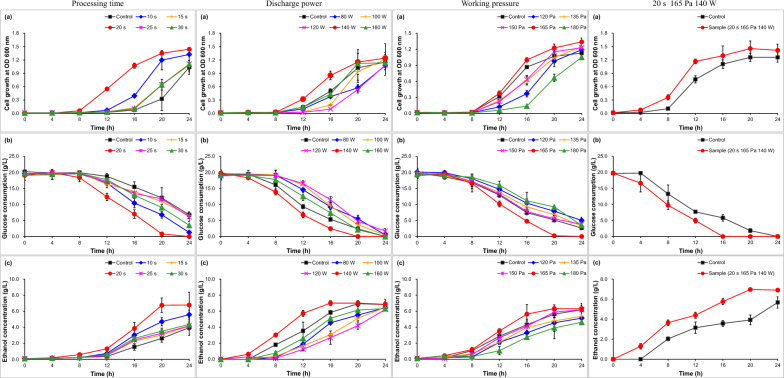
Ethanol fermentability of *Z. mobilis* ZM4 treated with cold plasma under the optimized parameters (20 s, 15 Pa, 140 W) in RM medium. **a** Cell growth; **b** glucose consumption; **c** ethanol concentration

It assayed the effect of different processing time of cold plasma on ethanol fermentability for *Z. mobilis* ZM4 at the default discharge power (135 Pa) and working pressure (120 W) (Fig. 3). Compared with the control (0.02), cell growth just for 20 s was increased by 212.28% at 8 h. Convincingly, cell growth of *Z. mobilis* ZM4 was separately increased by 188.89%, 43.06%, 2191.67%, 30.56% and 59.72% for 10, 15, 20, 25 and 30 s at 12 h (Fig. 3a). Glucose consumption was enhanced by 130.05% for 20 s at 8 h and by 95.32%, 85.39%, 352.50%, 65.14% and 29.38% for 10, 15, 20, 25 and 30 s at 12 h (Fig. 3b). Ethanol concentration was facilitated by 152.61% for 20 s at 8 h and by 102.83%, 41.51%, 264.00%, 32.26% and 69.73% for 10, 15, 20, 25 and 30 s at 12 h (Fig. 3c). Convincingly, ethanol fermentability for *Z. mobilis* ZM4 was enhanced after pretreated under all the processing time, and it achieved the most maximal ethanol fermentability at 20 s.

Furthermore, the effect of different discharge power of cold plasma on ethanol fermentability was examined at the default parameter of 135 Pa and 15 s (Fig. 3). Compared with the control (0.02 and 0.14), cell growth of *Z. mobilis* ZM4 for 140 W was separately increased by 21.67% at 8 h and 126.82% at 12 h (Fig. 3a). Compared with the control (3.18 g/L and 10.00 g/L), glucose consumption for 140 W was separately enhanced 46.08% at 8 h and 23.29% at 12 (Fig. 3b). Bioethanol concentration was also facilitated by 67.50% and 61.78% (Fig. 3c). In all, bioethanol fermentability was increased for 140 W after pretreated with cold plasma.

Bioethanol fermentability was also assayed at different working pressure of cold plasma at the default parameter (120 W and 15 s) (Fig. 3). Compared with the control, the increase just for 165 Pa was obtained by 20.15% for cell growth, 29.16% for glucose consumption and 22.98% for ethanol concentration at 12 h (Fig. 3a, b, c). It indicated that 135 Pa was the optimum parameter for working pressure.

In this work, the ethanol fermentability for *Z. mobilis* ZM4 was carried out under the above assayed optimum parameters of cold plasma (20 s, 165 Pa, 140 W) (Fig. 3). Cell growth was increased by 238.56% and 52.57% at 8 and 12 h, respectively (Fig. 3a), glucose consumption was increased by 50.85% and 23.43% (Fig. 3b) and ethanol concentration was increased by 79.48% and 39.74% (Fig. 3c). It indicated that cold plasma pretreatment brought about the facilitated ethanol fermentability for *Z. mobilis* ZM4.

### Genetic and transcriptional analysis for *Z. mobilis* ZM4 pretreated with cold plasma

In order to confirm the genetic changes and transcriptional landscapes derived from cold plasma pretreatment for *Z. mobilis* ZM4, it simultaneously carried out genome-based resequencing and transcriptomic sequencing.

**Fig. 4:**
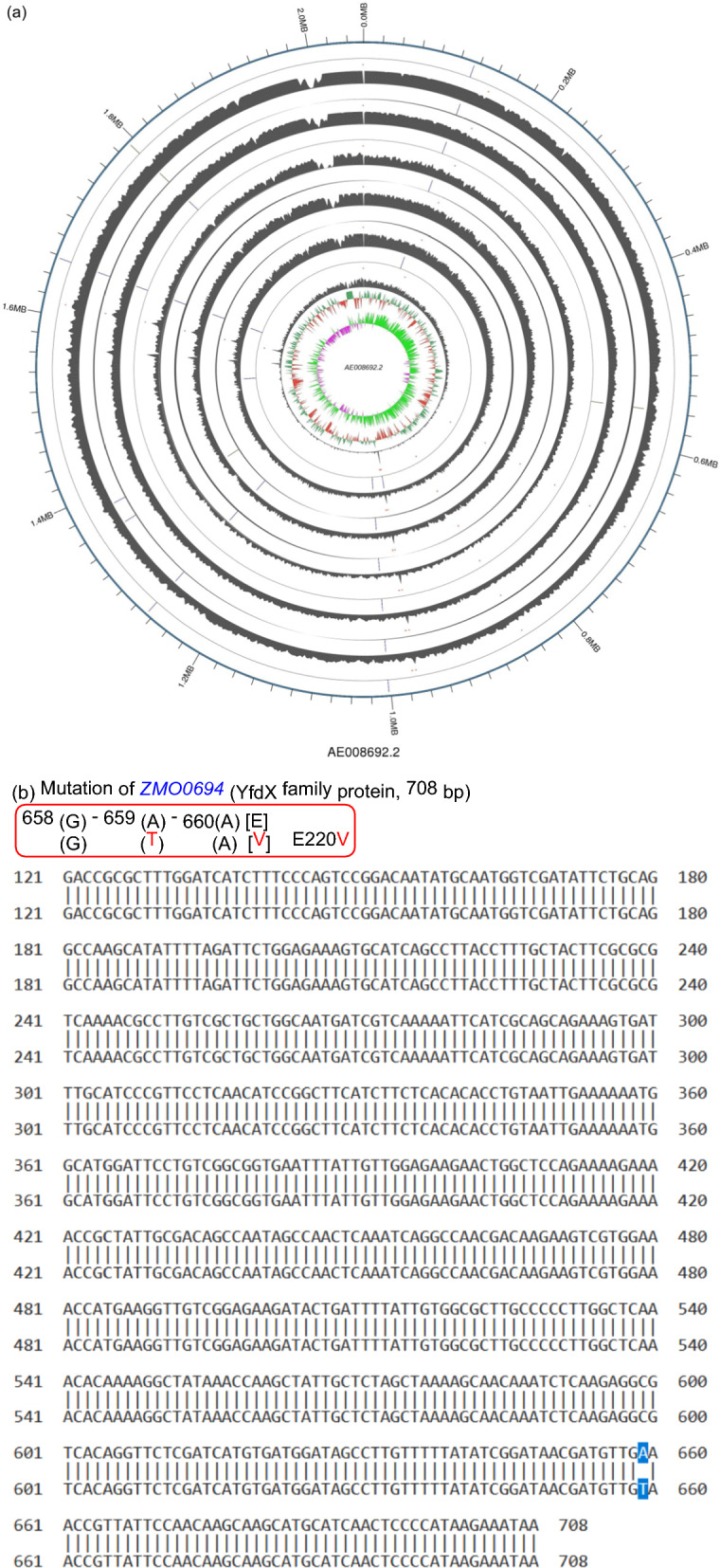

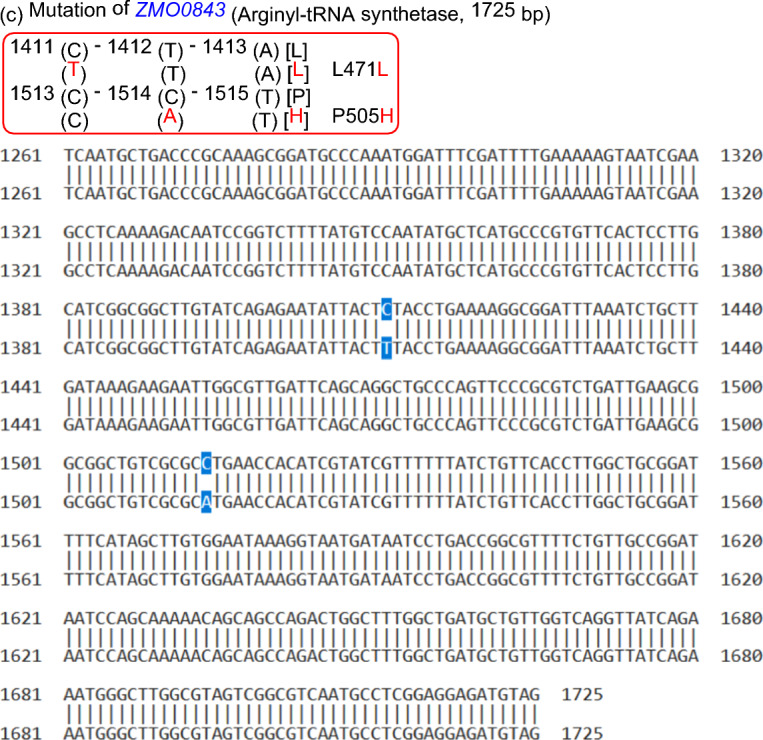
SNPs analysis for *Z. mobilis* ZM4 pretreated with cold plasma under the optimized parameters. **a** The whole genome mutation profile (WGMP). The outermost circle was the position coordinate axes of reference sequence. It showed InDel distribution, SNP numbers distribution, coverage depth of Reads, GC mol% content and GC skew value distribution of reference genome from the inside out. **b** Mutation of *ZMO0694*. **c** Mutation of *ZMO0843*

Figure 4 presents the whole genome mutation profile. SNPs analysis revealed that cold plasma brought about three point mutation (Fig. 4a), including *ZMO00694* (E220V) at the position of 691271 for genome and 659 for conserved gene sequence (Fig. 4b), *ZMO0843* (L471L) at the position of 849208 for genome and 1411 for conserved gene sequence and *ZMO0843* (P505H) at the position of 849311 for genome and 1514 for conserved gene sequence (Fig. 4c). It indicated that cold plasma really led to a certain mutations.

**Fig. 5:**
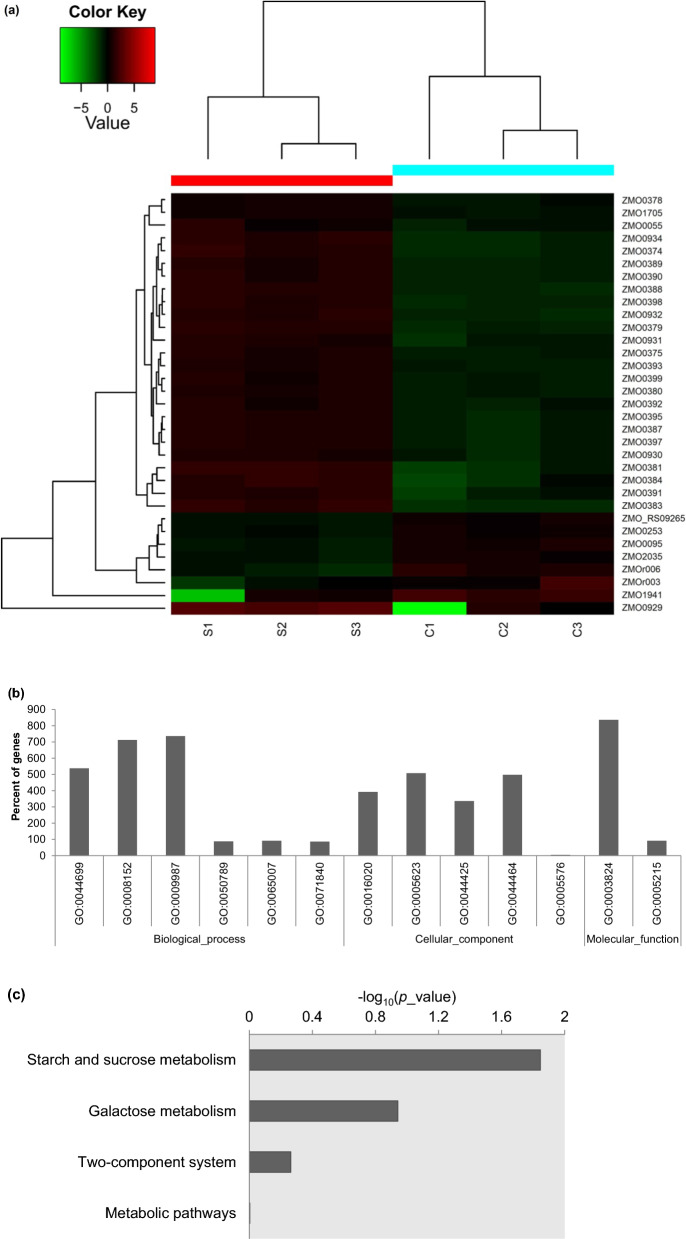
Transcriptomic analysis for *Z. mobilis* ZM4 pretreated with cold plasma using the optimized parameters. **a** The differentially expressed genes (DEGs); **b** GO analysis; **c** KEGG pathway analysis

Figure 5 shows the results of RNA-Seq sequencing. It was found that cold plasma pretreatment produced 33 DEGs (Fig. 5a). Except one gene without regulation information (*ZMOr007* ending 5S ribosomal RNA) and seven down-regulated DEGs, including *ZMO0253* and *ZMO_RS09265* (type I secretion outer membrane protein, TolC family), *ZMO2035* (conserved hypothetical replication initiator and transcription repressor protein), *ZMO0095* (hypothetical protein), *ZMOr003* and *ZMOr006* (16S ribosomal RNA) and *ZMO1941* (Type IV secretory pathway protease TraF-like protein), the other 25 up-regulated DEGs were involved with hypothetical protein, levansucrase, protein of unknown function DUF847/DUF81, phage terminase, large subunit, PBSX family, putative phage major head protein, conserved hypothetical protein, constituent protein and thioredoxin domain protein (Fig. 5a). Biological process was enriched single-organism process (GO:0044699), metabolic process (GO:0008152), cellular process (GO:0009987), regulation of biological process (GO:0050789), biological regulation (GO:0065007) and cellular component organization or biogenesis (GO:0071840). Cellular component was enriched membrane (GO:0016020), cell (GO:0005623), membrane part (GO:0044425), cell part (GO:0044464) and extracellular region (GO:0005576). Molecular function was enriched catalytic activity (GO:0003824) and transporter activity (GO:0005215) (Fig. 5b). For KEGG pathway analysis, it enriched starch and sucrose metabolism, galactose metabolism, two-component system and metabolic pathways in order (Fig. 5c). It revealed that cold plasma also contributed to a specific transcriptional profiling.

### The augmented aldehyde inhibitors tolerance and bioethanol fermentability for the mutant strain

**Fig. 6:**
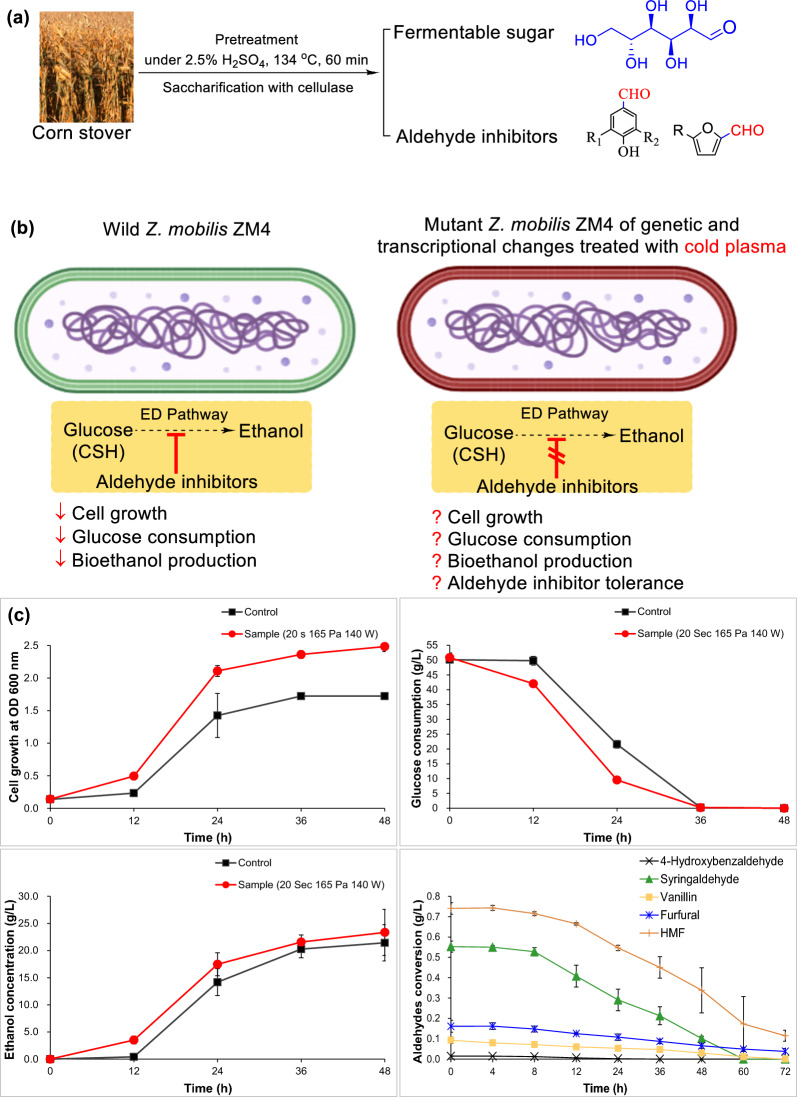
Ethanol fermentability in CSH for the pretreated *Z. mobilis* ZM4 using cold plasma. **a** The origin of glucose and aldehyde inhibitors in CSH; **b** speculation of ethanol fermentability and aldehyde inhibitor tolerance in CSH for the mutant; **c** validation of ethanol fermentability and aldehydes conversion tolerance in CSH for the mutant

Here, the resulting aldehyde inhibitors were derived from corn stover pretreated with dilute H_2_SO_4_ (Fig. 6a). Although it had confirmed that aldehyde inhibitors weakened ethanol fermentability in CSH (Fig. 1), it knew nothing of the enhancement of ethanol fermentability and aldehyde inhibitor tolerance for the mutant *Z. mobilis* ZM4 (Fig. 6b). Therefore, bioethanol fermentability for the mutated strain was further assayed in CSH (Fig. 6c). Compared with the control, cell growth of the *Z. mobilis* ZM4 was separately enhanced in 12 and 24 h by 112.59% and 47.95%, glucose consumption was facilitated by 144.85% and 33.32% and ethanol titer was increased by 455.31% and 51.31% (Fig. 6c). It proved that cold plasma treatment enhanced bioethanol fermentability for the mutant *Z. mobilis* ZM4 under the optimum parameters (20 s, 165 Pa and 180 W). Convincingly, 0.01 g/L 4-hydroxybenzaldehyde, 0.55 g/L syringaldehyde and 0.08 g/L vanillin were separately degraded by 87.22% at 24 h, 81.68% at 48 h and 87.82% at 60 h. The conversion rate of 0.16 g/L furfural and 0.75 g/L HMF was 76.31% and 84.55%, respectively, at 72 h. Therefore, it concluded that cold plasma pretreatment simultaneously conferred the robustness of aldehyde inhibitors tolerance and bioethanol fermentability for the mutant *Z. mobilis* ZM4.

## Discussion

With lots of desirable industrial strain biocatalysts brought about, *Z. mobilis* was always regarded as a robust ethanologen chassis, especially for bioethanol production using lignocellulosic materials. However, the lignocellulose-derived furanic aldehydes and phenolic aldehydes seriously blocked bioethanol accumulation for *Z. mobilis* [10, 29, 61–63].

The degradation of biomass during pretreatment may bring about the release of sugar monomers, furanic compounds, weak acids and phenolic compounds. The inhibitory intensity of the above inhibitors was usually different for the type of biorefinery strains [64–66]. For *Z. mobilis* ZM4, furanic aldehydes and phenolic aldehydes were more toxic than weak acids (acetic acid, formic acid, and levulinic acid) [10, 20, 62]. In this work, it confirmed that aldehyde inhibitors weakened the bioethanol fermentability in CSH. The previous study showed that 4-hydroxybenzaldehyde was the most toxic phenolic aldehyde for *Z. mobilis* ZM4 under the same concentration of 5 mM, followed by vanillin and syringaldehyde [10]. Here, it was illustrated that the toxicity of aldehyde inhibitors largely depended on their concentration in CSH for *Z. mobilis* ZM4. Here, inhibitory intensity of cell growth (mixed aldehydes > 0.75 g/L HMF > 0.08 g/L vanillin > 0.55 g/L syringaldehyde > 0.16 g/L furfural > 0.01 g/L 4-hydroxybenzaldehyde), glucose consumption (mixed aldehydes > 0.01 g/L 4-hydroxybenzaldehyde > 0.75 g/L HMF > 0.55 g/L syringaldehyde > 0.08 g/L vanillin > 0.16 g/L furfural) and ethanol production (mixed aldehydes > 0.75 g/L HMF > 0.080 g/L vanillin > 0.55 g/L syringaldehyde > 0.16 g/L furfural > 0.01 g/L 4-hydroxybenzaldehyde) were separately established. Obviously, mixed aldehydes were the most toxic for *Z. mobilis*, and thus well proved that the bioethanol fermentability was weakened by aldehyde inhibitors in CSH. What needs to be noted was that synergistic inhibition should be stressed, although *Z. mobilis* ZM4 was well tolerant furanic acids and phenolic acids [20]. For example, ethanol production was inhibited for the synergistic inhibition for the high concentration weak acid (acetic acid, formic acid and levulinic acid) and furan aldehydes (furfural and HMF) [67].

The effect of cold plasma on organism modification was tightly related to its generating device, the composition of the working gas, the distance from the cold plasma source to the samples and processing parameters including processing time, discharge power and working pressure [68]. Here, the above processing parameters were examined. The continuous increase for bioethanol production separately at 140 W of discharge power and 165 Pa of working pressure was possibly derived from the density of active substances from working gas, and thus was agreed with the documented data [69]. Interestingly, bioethanol fermentability was enhanced under all the assayed processing time for cold plasma pretreatment (10, 15, 20, 25, and 30 s). Combining the enhanced effect of discharge power and working pressure, it predicted that the accumulation of active substances might largely depend on the time span for cold pretreatment. On the other hand, it predicted that a certain span of time was necessary to process outer membrane structure of Gram-negative bacteria *Z. mobilis* ZM4 enough for the efflux of the small molecules (such as glucose). Therefore, the parameter of processing time might play a very vital role for *Z. mobilis* ZM4 to accumulate bioethanol.

Here, it was found that cold plasma pretreatment under the sub-lethal condition (20 s, 140 W and 165 Pa) produced a mutagenic effect on *Z. mobilis* ZM4, and thus was supported by the mutagenicity from cold plasma in bacteria in a parameter-dependent manner [43, 44, 70–72].

The impact of the plasma largely relied on the kind of an organism and its specific cell properties [73, 74]. In this study, cold plasma pretreatment produced a specific gene transcriptional profiling for *Z. mobilis* ZM4, and thus was consistent with the previous studies [51]. Some candidates from the screened 33 DEGs were presented as follows: (1) the two genes *ZMO0253* and *ZMO_RS09265* encoding type I secretion outer membrane protein (from TolC family) within the RND (Resistance-Nodulation-cell Division) efflux systems were differentially down-regulated after pretreated with cold plasma. Known as an ABC transporter system responsible for protein secretion without the cleavage of the signal sequence, outer membrane proteins were confirmed to involve with type I protein secretion and the efflux of the small molecules [75]. Outer membrane structure always made Gram-negative bacteria highly sensitive to peroxidation and more inclined to electrostatic disruption under cold plasma system [73], and thus well elucidated the down-regulation of outer membrane for *Z. mobilis* ZM4 after pretreated with cold plasma. (2) Of ionized gases with high energy of electrons and relatively low temperature of gas particles, cold plasmas could affect the exposed living cells [76]. Typically generated by various electrical discharges, cold plasma pretreatment itself could produce a stress of high energy and low temperature. It proved that low temperature could affect membrane fluidity, gene expression, protein synthesis and protein complexes stability in plants [77]. Therefore, the down-regulated gene *ZMO1941* (Type IV secretory pathway protease TraF-like protein) was also predicted as one of the contributors for its assayed gene function of stress tolerance [78]. (3) As the major components of ribosomes, ribosomal RNAs (rRNAs) responsible for their catalytic activity would undergo many modifications of ribosome biogenesis including transcription [79–82]. Here, it predicted that chilling stress derived from cold plasma might contribute to the transcriptional change for down-regulation of the two ribosomal genes *ZMOr003* and *ZMOr006*. (4) Levansucrase (EC2.4.1.10), a fructosyltransferase exoenzyme, was of with sucrose hydrolytic and levan biosynthetic activities. Specially, a certain amount of the levan could provide protection against diverse stresses protection [83–85]. Therefore, the two differentially up-regulated genes *ZMO0375* and *ZMO0374* encoding levansucrase were predicted to relate with stress resistance-derived from cold plasma pretreatment. (5) Here, *ZMO1705* encoding thioredoxins was up-regulated for *Z. mobilis* ZM4 after pretreated with cold plasma. Of a conserved active site motif (CGPC), thioredoxins (Trxs) could well perform stress tolerance through redox regulation of target proteins [86]. Convincingly, Trxs played a fundamental role in the stress response of cellular processes in microorganisms [87–90]. Most importantly, among 33 DEGs, the up-regulated genes were prevalent, and thus was predicted that cold plasma facilitated the transcriptional expression. On conclusion, cold plasma pretreatment conveyed stress tolerance for *Z. mobilis*, and thus was supported by some studies [91].

Convincingly, it confirmed that cold plasma pretreatment simultaneously reinforced aldehyde inhibitors tolerance and bioethanol fermentability for *Z. mobilis* ZM4 in CSH. It was proposed the genetic and transcriptional changes for the increased bioethanol fermentability achieved both in synthetic medium and CSH. However, most importantly, why did cold plasma pretreatment deliver stress tolerance of aldehyde inhibitors in CSH for the train *Z. mobilis* ZM4? The potential reasons were given as follows: (1) usually, mutations derived from the adaptation and evolution could make the bacteria fit and respond to a certain stress environment. Here, it predicted that the three identified point mutation derived from cold plasma pretreatment were possible in charge of the augmented aldehyde inhibitor tolerance and bioethanol fermentability for *Z. mobilis* ZM4, and thus was supported by YfdX family protein (*ZMO0694*) and arginine-tRNA ligase (argS) (*ZMO0843*) involved with stress resistance [92]; (2) *Z. mobilis* could efficiently accumulate bioethanol after defensed themselves against all the possible environmental threats, such as the toxic aldehyde inhibitors and cold plasma pretreatment. During this process, *Z. mobilis* cells would have to start a series of complex regulatory networks to overcome the adverse conditions and maintain their cell integrity. The stress resistance-related candidate genes might be responsible for the stress of aldehyde inhibitors, and thus be in agreement with the documented molecular mechanism of the stress tolerance [29, 93, 94].

The future study would be carried out as follows: firstly, the genetic stability of the mutant *Z. mobilis* from cold plasma pretreatment should be confirmed, and thus would provide a potential clue for the applications of the methodology in biorefinery fields. Secondly, it would assay the contribution of the three mutation sites to stress tolerance and bioethanol production. Last but not the least, the established gene and pathway datasets from deep sequencing would be investigated by gene engineering to promote the stress tolerance of the lignocellulose-derived inhibitors and the accumulation of biofuels and biochemicals from biomass for biorefinery strains.

## Conclusions

The work was focused on the enhancement of aldehyde inhibitors stress tolerance and bioethanol fermentability in CSH for *Z. mobilis* ZM4 pretreated with cold plasma. Compared with the control, a 6.99 g/L of bioethanol accumulation (equal to a 51.31% increase) at 24 h was acquired from *Z. mobilis* pretreated with cold plasma (20 s, 140 W and 165 Pa) in CSH. The specific genetic and transcriptional changes were also revealed for the augmented bioethanol production for *Z. mobilis* ZM4. This work would provide a strain biocatalyst for the efficient production of biofuels and other biochemicals in biorefinery fields.

## Data Availability

SNPs data of Genome re-sequencing are available with accession number of PRJEB61081 in European Variation Archive (EVA). RNA-Seq sequence data were deposited in the GEO database at NCBI. (https://www.ncbi.nlm.nih.gov/geo/query/acc.cgi?acc=GSE228538) in the GEO database at NCBI.

## Abbreviations

CSH: Corn stover hydrolysates
CAP: Cold atmosphere plasma
DEGs: Differentially expressed genes
GO: Gene Ontology
KEGG: Kyoto Encyclopedia of Genes and Genomes
SNPs: Single nucleotide polymorphisms
HMF: 5-Hydroxymethyl-2-furaldehyde
LTP: Low-temperature plasma
ARTP: Atmospheric and room temperature plasma
LVRTP: Low vacuum and room temperature plasma
NREL: National Renewable Energy Laboratory
RM: Rich medium
HTSeq: High-throughput sequencing
HPLC: High performance liquid chromatography
